# Antiseptic Activity of Ethnomedicinal Xuebijing Revealed by the Metabolomics Analysis Using UHPLC-Q-Orbitrap HRMS

**DOI:** 10.3389/fphar.2018.00300

**Published:** 2018-03-29

**Authors:** Lihua Zuo, Lin Zhou, Tanye Xu, Zhuolun Li, Liwei Liu, Yingying Shi, Jian Kang, Guanmin Gao, Shuzhang Du, Zhi Sun, Xiaojian Zhang

**Affiliations:** ^1^Department of Pharmacy, The First Affiliated Hospital of Zhengzhou University, Zhengzhou, China; ^2^Henan Key Laboratory of Precision Clinical Pharmacy, Zhengzhou University, Zhengzhou, China; ^3^Department of Rheumatology, The First Affiliated Hospital of Zhengzhou University, Zhengzhou, China

**Keywords:** UHPLC-Q-Orbitrap HRMS, metabolomics, Xuebijing, sepsis, anti-inflammatory

## Abstract

Xuebijing (XBJ) injection is an ethnomedicinal formula that has been widely used in the therapy of sepsis in China. However, the underlying theraputic mechanisms remain uninvestigated. In this research, a metabolomic method based on UHPLC-Q-Orbitrap HRMS was applied to make a holistic evaluation of XBJ on septic rats which were induced by the classical cecal ligation and puncture (CLP) operation. The plasma metabolic changes were profiled and evaluated by multivariate analytical (MVA) methods. In the results, a total of 41 differential metabolites were identified between CLP-operated group and sham-operated group, which were mainly involved in amino acid metabolism and lipid metabolism. After pathway analysis, it was finally discovered that the majority of the influenced metabolic pathways caused by sepsis mainly involved in energy metabolism, oxidative stress, and inflammation metabolism. When intervened by XBJ injection, 32 of the 41 disordered metabolites had been adjusted in reverse, which suggested that XBJ could mediate the abnormal metabolic pathways synergistically. In conclusion, the present study systematically investigated the efficacy and its underlying therapeutic mechanisms of XBJ on sepsis, while offering a new insight for the subsequent relevant exploration of other Chinese medicine at the same time.

## Introduction

Sepsis is a systemic inflammatory response syndrome (SIRS) that usually caused by polymicrobial bacterial infection (Brent, 2017). In septic patients, a mass of inflammatory factors (such as TNF-α, IL-1β, and IL-6) is produced, resulting in a more severe systemic inflammatory response that causes damage to Multiple Organ Dysfunction Syndrome (MODS) (Lu et al., 2017). Nowadays, the main drugs used to treat sepsis are glucocorticoids and antibiotics. The key point of treating sepsis is effective and rapid control of the inflammatory reaction. However, these treatments are only used to relieve the symptoms, which can't treat sepsis fundamentally and possess a lot of side effects usually. For instance, the antibiotic abuse could increase the risk of the emergence of drug-resistant bacteria and the multiple infections of patients. The lack of specific medicine has been the main reason for the high mortality. As a result, it's crucial to discover an appropriate drug for this disease. Fortunately, Traditional Chinese Medicine (TCM) may be a suitable candidate because it has been used to treat disease for over 2000 years in China and provided reliable health care to Chinese people long before the invention of the modern Western medicine (Li and Sang, 2017). TCM advocates combinatory therapeutic strategies, and the formulae containing different kinds of herbs can be used to increase efficacy and reduce the toxicity (Cheng et al., 2009; Li and Sang, 2017; Xu et al., 2017). Therefore, TCM will provide a huge source to treat different serious diseases.

Xuebijing (XBJ) injection is a TCM that consist of Salvia Miltiorrhiza, Angelica, Safflower, Radix Paeoniae Rubra, and Chuan Xiong (Zhang, 2008). Based on the five Chinese herbal medicine, XBJ has the functions of restraining exaggerated inflammatory response, which can develop a serious consequences for patients. Due to its the excellent therapeutic effect, XBJ injection had been approved by the State Food and Drug Administration (SFDA) of China, and now it is widely used in the treatment of SIRS, MODS, and sepsis in clinic (Shi et al., 2017). However, up to now, the current relevant research of XBJ injection treat on sepsis mainly focus on several single target points and pathway (Jiang et al., 2013; Chen et al., 2017). As TCM usually works by synergistic interactions and reflects a holistic dynamic efferts, it is inappropriate to focus on a single point in the study of XBJ injection treating sepsis. Therefore, a deeper exploration of the therapeutic mechanism of XBJ treating sepsis is needed urgently.

Metabolomics, as a downstream field of systems biology, is an integral technology for exploring the function of biological systems based on the global metabolite profiles of drug treatments (Cheng et al., 2017). Metabolomics mainly focus on the whole metabolic networks rather than on individual metabolites, and this characteristic is in highly accordance with the synergistic interactions properties of TCM, which makes it particularly suitable for investigating underlying mechanism of Chinese herbal medicines (Qi et al., 2014). Therefore, metabolomics has been an appropriate method to evaluate the efficacy and explore the mechanism of TCM on complex disease. While, in consideration of the matrix complexity of the biological sample, the detection instrument will be an important factor that influences the metabolomics analysis result. Fortunately, a novel orbitrap high resolution mass spectrometry analytical platform, possessing higher mass resolution and mass accuracy, wider dynamic range, and better sensitivity, could provide strong technical support for metabonomic researches in complex biological and TCM samples. Therefore, in this study, a metabolomics method based on the ultra performance liquid chromatography-quadrupole/orbitrap high resolution mass spectrometry (UHPLC-Q-Orbitrap HRMS) technology was developed for exploring the therapeutic mechanism of XBJ. To our knowledge, it's the first time to investigate the therapeutic mechanism of XBJ treating sepsis based on the metabolomics method, and it is expect to offer a new insight for the subsequent relevant exploration of other Chinese medicine.

## Materials and methods

### Instrument, reagents, and materials

UHPLC-Q-Orbitrap System: Ultimate 3000 UHPLC (Dionex, USA), Q Exactive high resolution mass spectrometry (Thermo Fisher Scientific, USA); ACQUITY UHPLC® BEH C_18_ (50 × 2.1 mm, 1.7 μm) chromatographic column, (Waters, USA); BX7200HP Desktop ultrasonic cleaner, (ShangHai CIMO Medical Instrument co., Ltd. China); AL104 balance with 0.0001 accuracy, (Mettler Toledo, Switzerland); ELISA (Bio-rad, USA); Plate Washer(Thermo Scientific, USA).

TNF-α, IL-1β, and IL-6(R&D, USA); HPLC-grade methanol and acetonitrile were purchased from Fisher Scientific (Fair Lawn, NJ, USA); Formic acid was HPLC grade and obtained from Aladdin Industrial Co., Ltd. (Shanghai, China); and the other chemicals were of analytical reagent grade. Ultra-pure water (18.2 MΩ) was prepared by the Milli-Q purification system (Millipore, Shanghai, China); XBJ injections were purchased from Tianjin Chase Sun Pharmaceutical Co., Ltd. (Tianjin, China) and all solutions were filtrated from 0.22 μm pore size filters.

Male Sprague-Dawley (SD) rats (2 months old, 250–300 g) were purchased from experimental animal center of henan province (Zhengzhou, China). All animals were fed to adapt to the environment under the controlled temperature (20 ± 2°C), humidity(60 ± 5%) and 12 h light/12 h dark cycle for 1 week before the experiment.

### UHPLC-MS/MS system conditions

An ultra high performance liquid chromatography (UHPLC) system was used to separate the metabolites in the plasma, and 5 μL aliquot from the per sample was injected into a ACQUITY UHPLC® BEH C_18_ column maintained at 40°C. Phase A was acetonitrile and phase B was water containing 0.1% formic acid. The gradient elution was as follows at a flow rate of 0.2 mL/min: 0~0.5 min, 5% A; 0.5~1.0 min, 5%~60% A; 1.0~7.0 min, 60%~80% A; 7.0~9.0 min, 80~100% A; 9.0~11.0 min, 100% A; 11.0~13.0 min, 5% A.

A Q Exactive high resolution mass spectrometry was tandem to the UHPLC system using an electrospray ionization (ESI) ion source. The temperature of the auxiliary gas, ion source and capillary were 300°, 350°, and 320°, respectively, the flow rate of the auxiliary gas was 10 μL/min. Samples were respectively tested in the positive and negative modes by full scan/ddms2 scan patterns from 80 to 1200 m/z at the mass resolving power of 17,500 in MS/MS. The gradient collision energy was at 20, 30, and 40 eV. The spray voltage and the sheath gas flow rate were set to 3.50 kV and 40 μL/min for the positive mode and 2.80 kV and 38 μL/min for the negative mode. Regarding the sequence of analysis, all samples were randomized. Blank samples containing barely the solvent were added after every 20 samples, aiming to elute residuals or other impurities which were retained by the chromatography column in the previous spectra records.

### Cecal ligation and puncture (CLP) model of sepsis

The animal experiments were approved by Animal Ethics Committee of the first affiliated hospital of Zhengzhou University and conformed to the ethical use of animals of the National Institute of Health guidelines. The CLP procedure was performed as the Nature Protocols described previously (Rittirsch et al., 2009). To put it most simple, rats were anesthetized by ketamine (80 mg/kg, Yuhan Corporation, Seoul, Republic of Korea) and xylazine (15 mg/kg, Bayer, Germany), a midline laparotomy was performed with an incision less than 3 cm. In order to maintain the intestinal continuity, the cecum was ligated tightly just below the ileocecal valve with a 2-0 silk. The antimesenteric surface of the cecum was punctured with an 18-gauge needle between the ligation and terminal, then the cecum was gently compressed until fecal contents was extruded. The bowel was then returned to the abdomen and the incision was sutured. After the operation, all rats were resuscitated with saline (0.3 mL/Kg), given subcutaneously. The sham-operated groups were given a laparotomy, and the cecum was operated but not ligated or punctured. Then all animals were returned to their cages with free access to food and water.

### Grouping and animal treating

Eighteen rats were divided into three groups equally and randomly. The experimental grouping was as follows: CLP group(C group): rats received tail vein injection of saline solution (0.4 mL/100 g) at 0 and 6 h after CLP operation; Sham group(S group): rats received tail vein injection of saline solution (0.4 mL/100 g) at 0 and 6 h after sham operation; CLP+XBJ group(X group): rats received tail vein injection of XBJ (0.4 mL/100 g) at 0 and 6 h after CLP operation.

### Sample collection

Blood samples were collected via the abdominal artery of rats from three different groups at 12 h after surgery. Six rats in each group were used for blood sampling and in order to collect the sample, rats were anesthetized by ketamine (80 mg/kg, Yuhan Corporation, Seoul, Republic of Korea) and xylazine (15 mg/kg, Bayer, Germany). A laparotomy was made and the abdominal artery of the rat was exposed after shifting the intestines over to the left side. The blood was obtained after inserting a 21-gauge needle combined a 10 mL syringe into the abdominal artery and drawing the blood slowly. Five microliters plasma was collected after centrifuging the samples stored in heparinization EP tube (3500 rpm, 4°C for 10 min).

### Model validation and the evaluation of the xuebijing treatment

The mechanisms of sepsis is complex and primarily mediated by cytokines, and it is now well-established that the activation of cytokine network is caused by polymicrobial bacterial infection (DiPiro, 1997). As a kind of cytokines, TNF-α is a potent inducer that play an important role in metabolism and is deemed to be the first proinflammatory cytokine that is released, followed by others including IL-1β and IL-6. Moreover, IL-6 seems to work synergistically with these other cytokines (Thijs and Hack, 1995; Hack et al., 1997; Englert and Rogers, 2016). During the sepsis, the serum TNF-α, IL-1β, and IL-6 are mediators of inflammation and usually used in the diagnosis and the evaluation of the therapeutic efficiency (Kurt et al., 2007).

As is described above, sepsis is highly associated with the inflammation factor and TNF-α, IL-1β, IL-6 could be the index of sepsis pathology. Therefore, C group would reflect a high level of inflammatory cytokines and X group would show an opposite trend in this study. Levels of the TNF-α, IL-1β, and IL-6 were measured using Enzyme-Linke Immuno Sorbent Assay (ELISA) kits purchased from R&D company (USA) according to the manufacturer's instructions. In this way, we could validate the sepsis model was successful or not, and whether the XBJ could play an anti-inflammatory effect on sepsis.

### Sample preparation

All plasma samples were stored at hypothermia refrigerator (−80°C) until the sample preparation. After thawing the samples on ice, 20 μL plasma of each sample was mixed to form the pooled plasma. And then the pooled plasma was subpackaged into equal aliquots as the quality control (QC) samples. The plasma (100 μL) sample was spiked with 300 μL methanol solution (containing 0.1 mg/mL L-2-chlorophenylalanine and ketoprofen as the internal standard). After vortexing for 3 min, the mixture was centrifuged at 13,300 rpm at 4°C for 15 min. The supernatant was extracted and transferred to an auto-sampler vial for analysis. The order of sample analysis was randomized. Several QC samples were analyzed until the system was stable. And in the sequence of sample analysis, the QC samples were inserted every five samples.

### Data processing and statistical analysis

All data was acquired and processed using Thermo Xcalibur™ software (Version 3.0, Thermo Scientific). And then the peak calibration, peak matching and peak alignment would be performed by the SIEVE software (Version 2.2, Thermo Scientific) to extract the information. Concretely, for alignment, the retention time width was set to 0.1 min with the mass width of 5 ppm. Ion peaks were filtered with an intensity threshold of 1,000,000 for both positive and negative modes. Normalization and scaling of the ion peaks were then performed. The data result sets containing all the m/z value, retention time and ion peak area of each sample, which corresponds to the concentration of certain metabolite, were exported to the multivariate statistical software SIMCA (version 14.0, Umetrics, Umea, Sweden) for the subsequent principal component analysis (PCA) and orthogonal partial least square discrimination analysis (OPLS-DA), then the preliminary discriminating metabolites would be selected by plotting the data set of all the discriminators in a Variable Importance in Projection (VIP) plot and S-plot. In order to further screening the significant variables between different groups, a student's *t*-test and fold change value of all the detected peaks were carried out using the SPSS 21.0 software (IBM, USA). Finally, metabolites which were statistically significant and responsible for the separation between two groups were selected and identified. A of Heat Map was generated with these screened metabolites by the Multi Experiment Viewer (Mev, Version 4.9.0, Dana-Farber Cancer Institute, MA, USA) to show the change trend. The values were expressed as mean ±SEM, and the histogram of some data were drawn using the Origin software (Version 9.2, OriginLab, Northampton, USA).

## Results

### The inflammatory cytokines level of the different group

**Figure 1:**
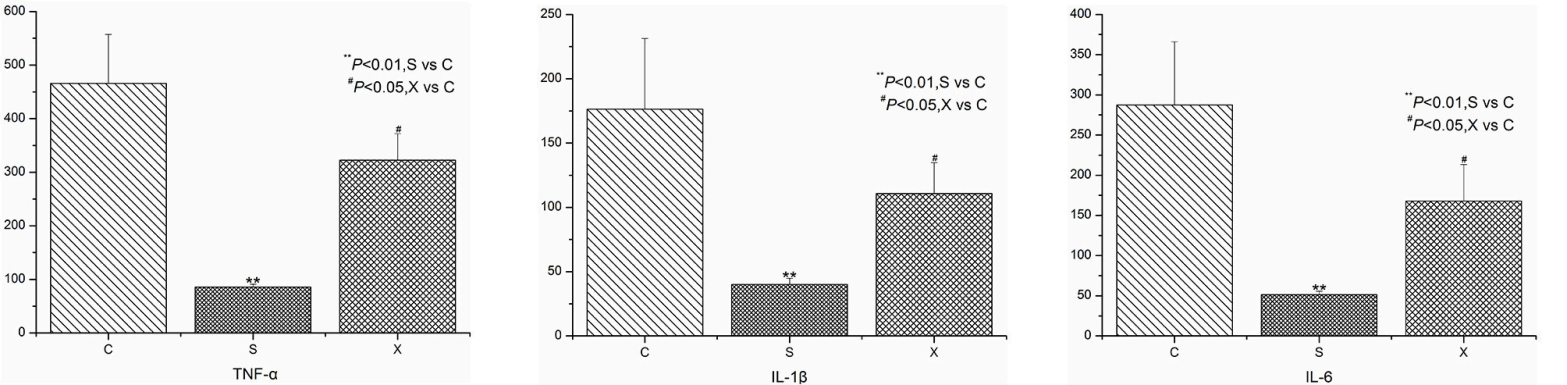
The concentration level of IL-1β, TNF-α, and IL-6 in different groups.

The experimental operating was in strict accordance with the standard operating procedure of the kit instructions. Finally, the *r* values of the standard curves were 0.995, 0.997, 0.998 for IL-1β, TNF-α, and IL-6, respectively, indicating a good and credible result. The levels of the inflammatory cytokines in different group are expressed in Figure 1. From the picture, it is clear that the IL-1β, TNF-α, and IL-6 show a higher level in C group than the S group. The result was considered significant in statistics (*P* < 0.01), illustrating that the sepsis model was successful. Meanwhile, the inflammatory cytokines level in X group was obviously lower than the C group (*P* < 0.05), providing a powerful evidence that the XBJ injections had an outstanding anti-inflammatory effect on sepsis.

### Pretreatment of peaks and assessment of QC samples

**Figure 2:**
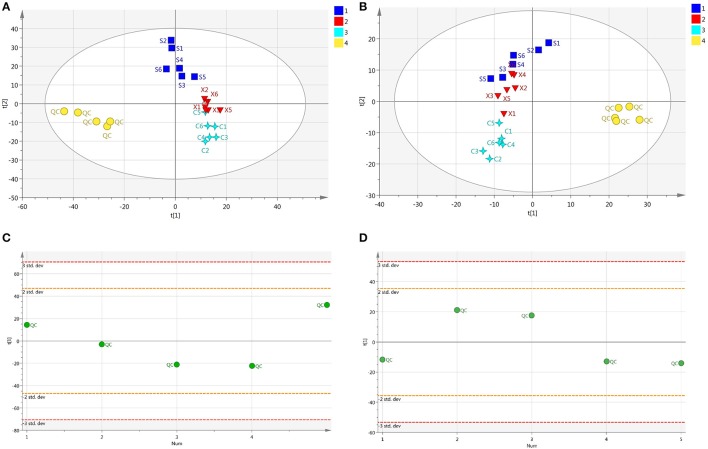
**(A)** The PCA plot of QC and samples in positive ion mode, **(B)** The PCA plot of QC and samples in negative ion mode, **(C)** The scatter distribution plot in the first principal components in positive ion mode, **(D)** The scatter distribution plot in the first principal components in negative ion mode.

Nine QC samples were analyzed before samples to test the stability and the repeatability of the equipment. 1,359 ion peaks in positive and 787 in negative combined with their areas were captured in mixed QC samples by the alignment software. After normalized, relative standard divergence (RSD) of every ion peak in all QC samples was calculated. The results showed that ion peaks with RSD <0.30 occupied 81.1% of total peak area in positive mode and 92.1% in negative mode, indicating that the captured spectra was relatively stable and the obtained data were reliable for statistical analysis. As for samples data, the ion peaks would be excluded when their RSDs were higher than 30% in QC samples before following analysis. Furthermore, peaks which the zero area occupied more than 20% of the integrated peak area would be also eliminated. Then, a mass of significative ion peaks were reserved in positive or negative modes, respectively. All the peaks were included for the QC samples and samples, and a PCA for all the samples was performed. The QC samples were observed to stay together in positive and negative ion mode respectively which was opposite to samples, and shown a good stability of the experiment in Figures 2A,B. Furthermore, in order to investigate the deviance in different QC samples, a scatter distribution plot in the first principal components was displayed in Figures 2C,D, and the result showed that all the QC samples distributed in the ranges of 2SD, indicating the experiment operations were consistent and the instrument system was stable.

### Screening and identification of potential biomarker

**Figure 3:**
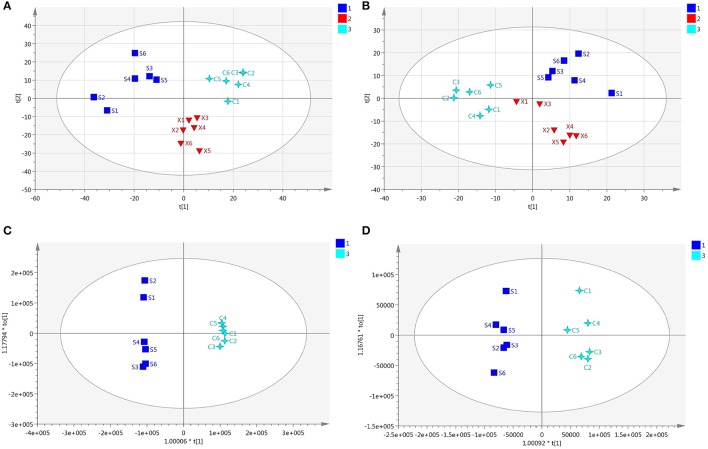
**(A)** The PCA plot of the different groups in positive ion mode, **(B)** The PCA plot of the different groups in negative ion mode, **(C)** The OPLS-DA plot of the different groups in positive ion mode, **(D)** The OPLS-DA plot of the different groups in negative ion mode.

**Figure 4:**
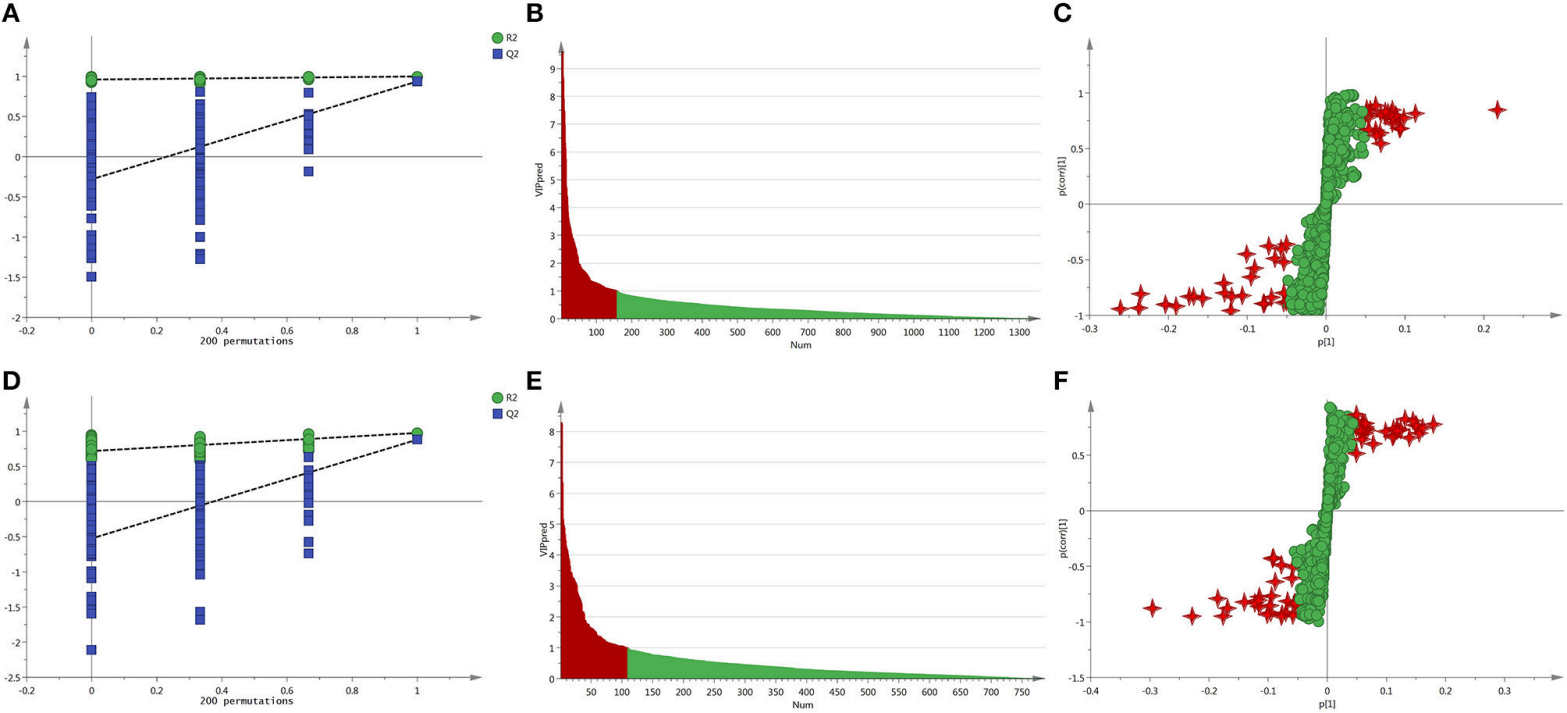
**(A)** The permutations test of the C vs. S group in positive ion mode, **(B)** The VIP plot of the C vs. S group in positive ion mode, **(C)** The S-plot of the C vs. S group in positive ion mode, **(D)** The permutations test of the C vs. S group in negative ion mode, **(E)** The VIP plot of the C vs. S group in negative ion mode, **(F)** The S-plot of the C vs. S group in negative ion mode.

Every sample has two data sets established by positive and negative mode, and they would be displayed at the same time. After normalized and centered, the ion peak areas of the different groups except the QC samples were then submitted to the SIMCA software. In PCA model, after automatic fitting, an apparent separation between different groups has been displayed in the scatter plot (shown in Figures 3A,B), and the plot showed that the organism state of the rats was completely different in different groups. In order to explore the reasons that led to the state differences, an OPLS-DA model was established, and the separation between C and S group was very clearly presented with excellent values in the scatter plot of the model (shown in Figures 3C,D). In addition, a permutation of 200 times was used to validate the OPLS-DA model and it showed a good result without overfitting in Figures 4A,D. From the established OPLS-DA model, VIP value, and S-plot could help to find the potential metabolites that responsible for the different organism state. In the VIP plot, the value greater than 1.0 was considered to have significance and in the S-pot, the metabolites distributed away from the origin had important roles (shown in Figures 4B,C,E,F).

**Figure 5:**
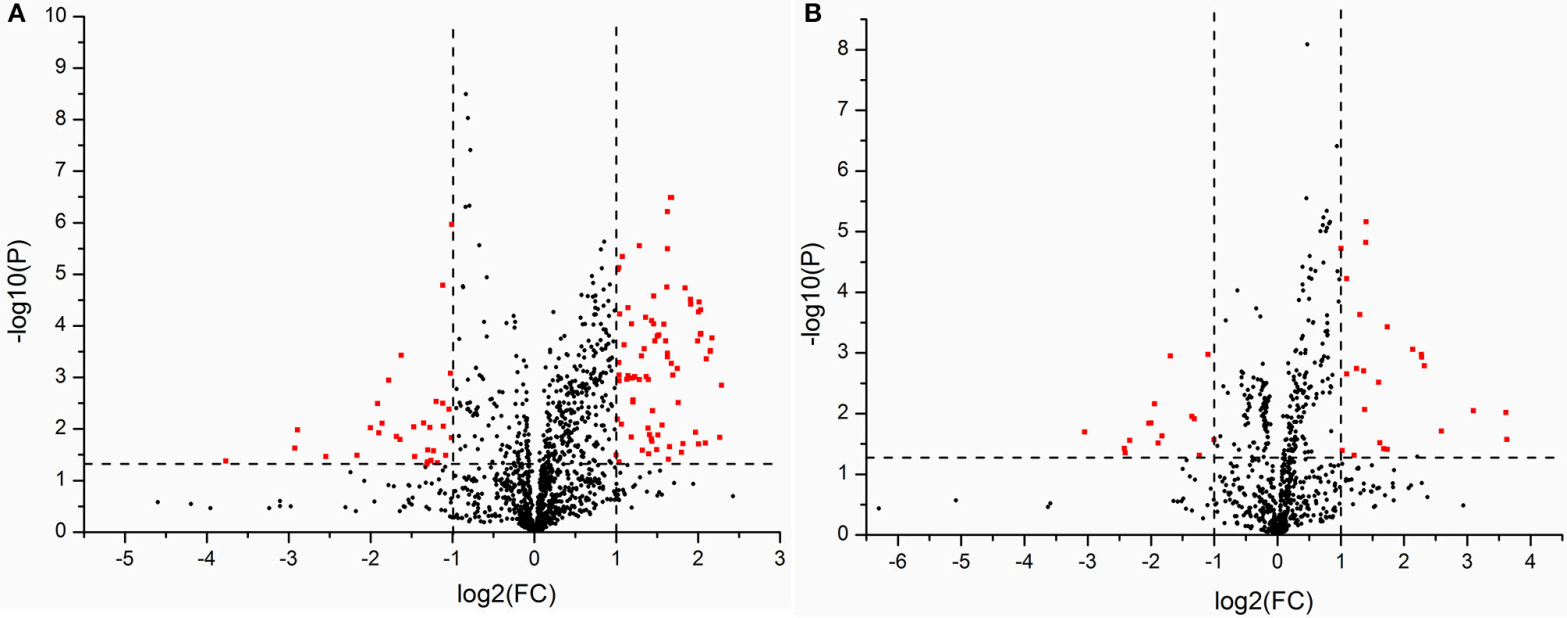
**(A)** The volcano plot of the C vs. S group in positive ion mode, **(B)** The volcano plot of the C vs. S group in negative ion mode.

Moreover, student's *t*-test and fold change values were also calculated to ensure the detected metabolites had statistical significance and obvious concentration changes. The metabolites whose *P*-values were greater than 0.05 or the fold changes were small would be excluded, and the others which showed a lower *P* and higher fold change value would be kept. A volcano plot containing the *P*-values of student's *t-*test and fold change values was performed between two groups to discover the final metabolites in Figures 5A,B. In the volcano plot, the red origin represented the metabolites whose *P* values were below 0.05(–log_10_*P* > 1.30) and the fold change values were greater than 2 or smaller than 0.5(log_2_FC ≥ 1 or log_2_FC ≤ −1). By these ways, several potential biomarkers were finally found that distinguished the C croup and S group.

The identification work of the reserved metabolites was realized by searching the obtained exact molecular mass data (m/z) in METLIN, HMDB, M/Z cloud, and the database established by ourselves. In the end, 41 metabolites that led to the main difference between C group and S group were identified in positive and negative mode. Among the identified substances, 32 of them were reversibly adjusted by XBJ injections in X group and even reached the level in the S group (shown in Table 1).

**Table 1:** Different metabolites identified between C group vs. S group and adjusted in X group.

**No**	**m/z**	**Metabolites**	**Formula**	**VIP**	**Fold change(C/S)**	**Adjusted by Xuebijing Fold change(X/C)**	**Pathway involved**	**Δppm**
1	116.071	Proline	C_5_H_9_NO_2_	4.48	0.36↓^*^	1.72↑^*^	Proline metabolism	1.076
2	150.058	L-Methionine	C_5_H_11_NO_2_S	2.89	0.43↓^*^	1.63↑^*^	Methionine metabolism	−0.039
3	229.154	Prolylleucine	C_11_H_20_N_2_O_3_	1.14	0.37↓^*^	3.18↑^*^	Prolylleucine metabolism	−2.614
4	132.102	Leucine	C_6_H_13_NO_2_	9.52	0.37↓^*^	1.76↑^*^	Leucine metabolism	−0.267
5	146.165	Spermidine	C_7_H_19_N_3_	1.69	0.36↓^*^	1.90↑^*^	Arginine and proline metabolism	−2.081
6	145.061	L-Glutamine	C_5_H_10_N_2_O_3_	1.07	0.50↓^*^	1.63↑^*^	Glutamate metabolism	0.905
7	160.037	4-Methylene-L-glutamate	C_6_H_9_NO_4_	1.49	0.20↓^*^	3.74↑^*^	Glutamate metabolism	−0.839
8	586.315	N-Acetyl-D-glucosaminyldiphosphodolichol	C_23_H_43_NO_12_P_2_	1.16	0.30↓^*^	1.79↑^*^	N-Glycan biosynthesis	0.433
9	123.055	Nicotinamide	C_6_H_6_N_2_O	1.41	0.49↓^*^	1.53↑^*^	Nicotinate and nicotinamide metabolism	0.736
10	138.055	Methyl nicotinate	C_7_H_7_NO_2_	1.05	0.49↓^*^	1.86↑^*^	Nicotinate and nicotinamide metabolism	−0.688
11	581.320	Stearyl monoglyceridyl citrate	C_28_H_54_O_12_	1.63	0.41↓^*^	1.75↑^*^	Lipid metabolism	4.398
12	269.226	anhydroretinol	C_20_H_28_	1.27	0.49↓^*^	0.91	Vitamin A metabolism	−1.699
13	335.223	Prostaglandin B1	C_20_H_32_O_4_	1.10	0.49↓^*^	1.57↑^*^	Arachidonic acid metabolism	3.264
14	319.226	15-Deoxy-Δ12,14-prostaglandin A1	C_20_H_30_O_3_	1.36	0.44↓^*^	0.87	Arachidonic acid metabolism	−2.197
15	218.139	Propionyl carnitine	C_10_H_19_NO_4_	1.07	2.05↑^*^	0.42↓^*^	Fatty acid metabolism	−0.755
16	400.342	Palmitoyl carnitine	C_23_H_45_NO_4_	1.36	2.03↑^*^	0.77	Fatty acid metabolism	−1.387
17	428.373	Stearoyl carnitine	C_25_H_49_NO_4_	1.02	2.77↑^*^	0.65↓^*^	Fatty acid metabolism	−2.557
18	357.278	Tetracosahexaenoic acid	C_24_H_36_O_2_	1.79	2.39↑^*^	0.86	Polyunsaturated fatty acid metabolism	−3.490
19	101.023	2-oxobutanoic acid	C_4_H_6_O_3_	1.02	0.50↓^*^	1.30	Propanoate, Cysteine and methionine metabolism	0.885
20	103.039	3-hydroxybutanoic acid	C_4_H_8_O_3_	4.33	2.46↑^*^	0.48↓^*^	Butanoate metabolism	−2.141
21	124.006	Taurine	C_2_H_7_NO_3_S	1.02	0.49↓^*^	1.55↑^*^	Taurine and hypotaturine metabolism	0.240
22	391.283	12-Ketodeoxycholic acid	C_24_H_38_O_4_	1.14	2.15↑^*^	0.64↓^*^	Primary bile acid biosynthesis	−2.214
23	407.280	Cholic acid	C_24_H_40_O_5_	3.70	3.84↑^*^	0.65↓^*^	Primary bile acid biosynthesis	1.889
24	212.002	3-Indoxyl sulfate	C_8_H_7_NO_4_S	1.72	0.47↓^*^	1.45	Tryptophan metabolism	1.581
25	583.336	Cholic acid glucuronide	C_30_H_48_O_11_	1.31	0.47↓^*^	2.09↑^*^	Pentose and glucuronate interconversions	1.974
26	105.110	Choline	C_5_H_14_NO	1.14	2.10↑^*^	0.51↓^*^	Glycerophospholipid metabolism	−4.379
27	476.278	LysoPE(18:2)	C_23_H_44_NO_7_P	1.03	0.48↓^*^	1.56↑^*^	Phospholipid metabolism	1.710
28	482.324	LysoPE(18:0)	C_23_H_48_NO_7_P	1.17	0.49↓^*^	1.63↑^*^	Phospholipid metabolism	−1.111
29	526.315	LysoPE(22:5)	C_27_H_46_NO_7_P	1.00	0.50↓^*^	1.05	Phospholipid metabolism	1.452
30	520.339	LysoPC(18:2)	C_26_H_50_NO_7_P	7.04	0.46↓^*^	1.29	Phospholipid metabolism	−0.914
31	542.321	LysoPC(20:5)	C_28_H_48_NO_7_P	1.29	0.30↓^*^	1.83↑^*^	Phospholipid metabolism	−3.671
32	546.352	LysoPC(20:3)	C_28_H_52_NO_7_P	1.66	0.43↓^*^	1.95↑^*^	Phospholipid metabolism	−1.804
33	550.385	LysoPC(20:1)	C_28_H_56_NO_7_P	1.04	0.47↓^*^	1.35	Phospholipid metabolism	−1.773
34	568.338	LysoPC(22:6)	C_30_H_50_NO_7_P	3.21	0.49↓^*^	1.93↑^*^	Phospholipid metabolism	−1.576
35	494.323	LysoPC(16:1)	C_24_H_48_NO_7_P	1.18	0.46↓^*^	2.36↑^*^	Phospholipid metabolism	−2.257
36	510.355	LysoPC(17:0)	C_25_H_52_NO_7_P	1.08	0.50↓^*^	1.59↓^*^	Phospholipid metabolism	−2.284
37	330.336	Dihydroceramide	C_19_H_39_NO_3_	1.57	0.49↓^*^	1.65↑^*^	Sphingolipid metabolism	−4.089
38	352.290	Sphingosine 1-phosphate	C_16_H_34_NO_5_P	1.01	0.50↓^*^	1.57↑^*^	Sphingolipid metabolism	−4.917
39	703.574	Palmitoyl sphingomyelin	C_39_H_79_N_2_O_6_P	3.27	2.07↑^*^	0.58↓^*^	Sphingolipid metabolism	−2.119
40	112.051	cytosine	C_4_H_5_N_3_O	1.10	0.49↓^*^	1.50↑^*^	pyrimidine metabolism	0.014
41	179.055	D-(+)-Glucose	C_6_H_12_O_6_	1.89	0.47↓^*^	1.81↑^*^	glucose metabolism	1.762

* *The values have statistical significance (P < 0.05); ↑ The metabolites were up-regulated; ↓ The metabolites were down-regulated*.

### Metabolite data analysis

**Figure 6:**
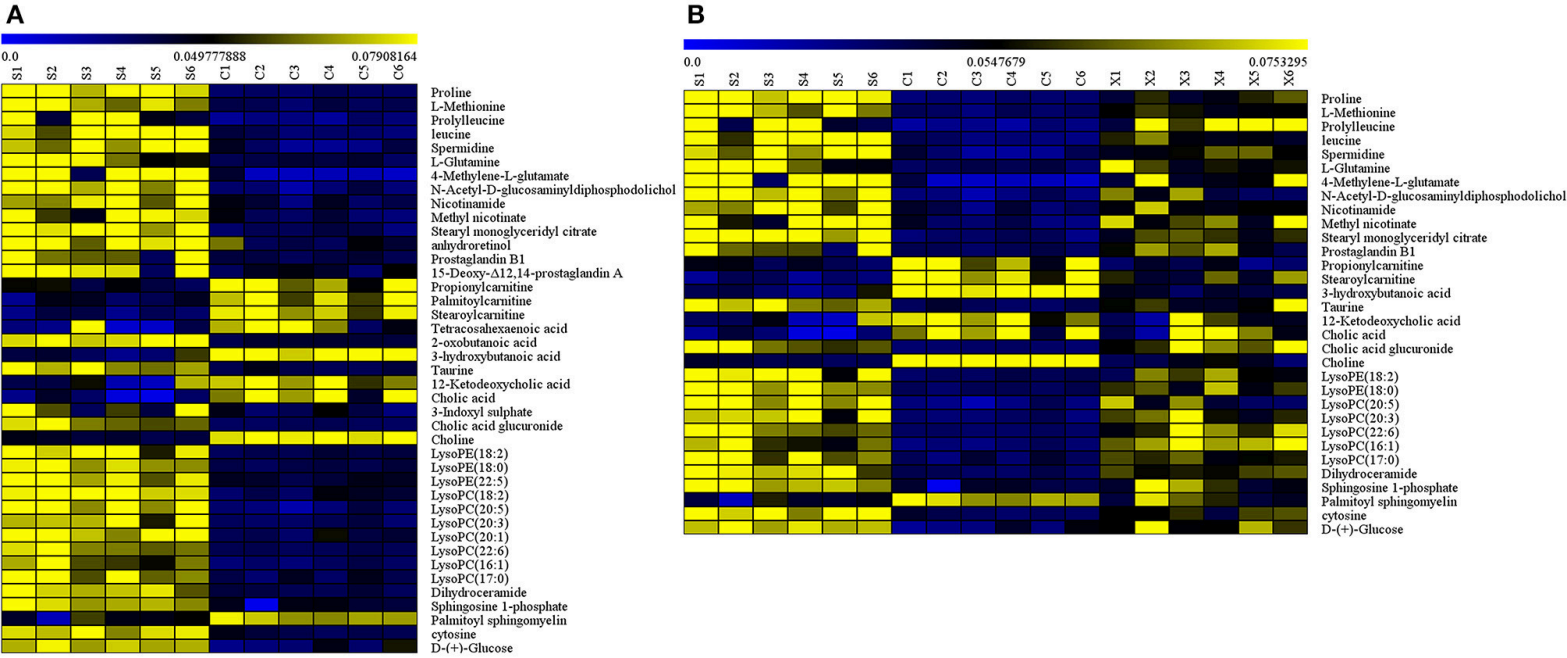
**(A)** The heat map of the disordered metabolites in C vs. S group, **(B)** The heat map of the metabolites adjusted by XBJ injection in C, S, and X group.

A heat map was then applied to reveal the change direction of the selected metabolites discriminators by Mev software (shown in Figures 6A,B). From the heat map (Figure 6A), an obvious demarcation line was found between C group and S group, showing that the metabolites had occurred a great changes in the sepsis disease. While, Figure 6B showed that most of the metabolites changed in C group had reversibly adjusted in X group by XBJ injections. As a result, XBJ injections had played a huge role in regulating the metabolic pathways.

### Pathway analysis and the correlation network

**Figure 7:**
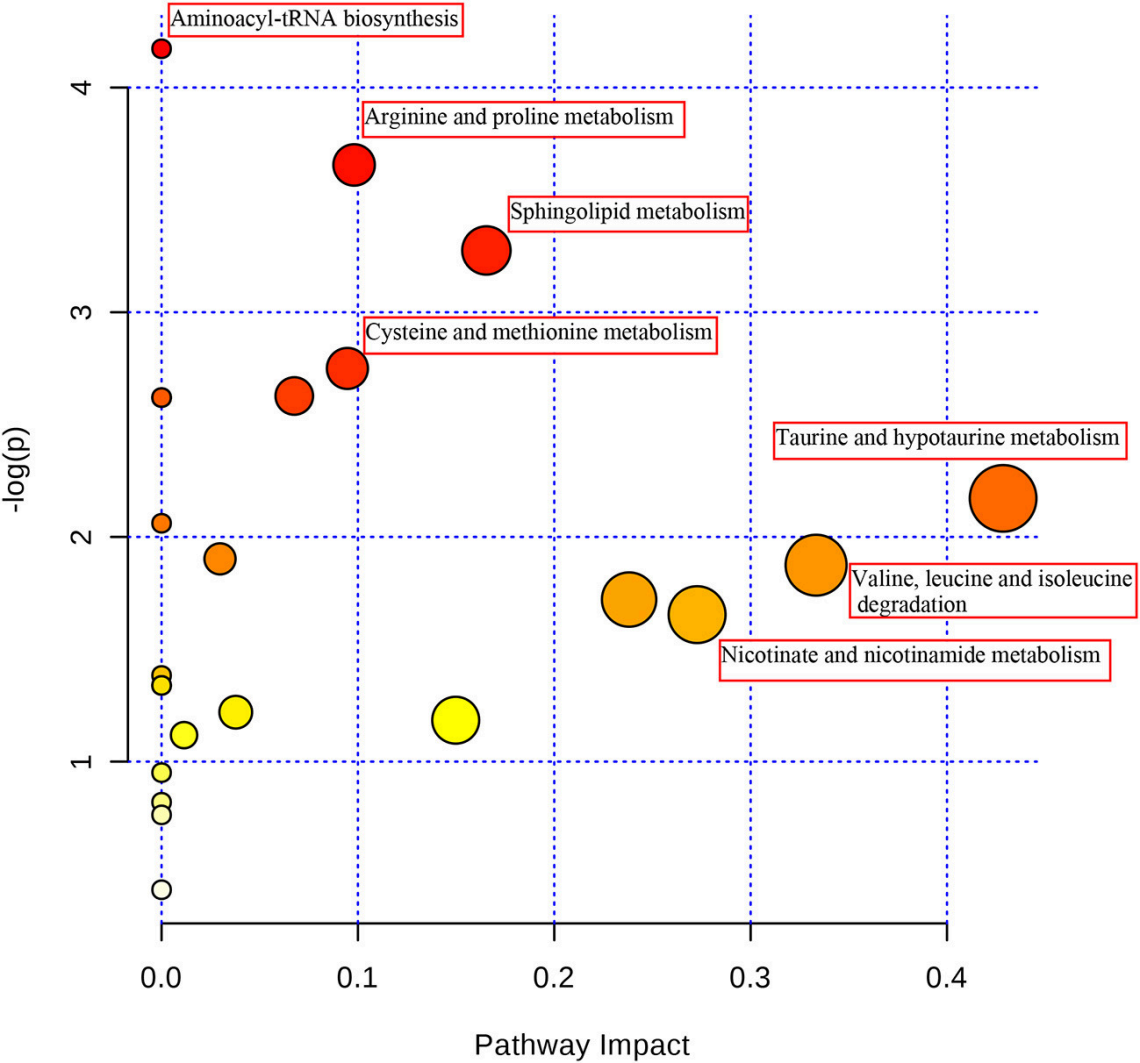
The pathway analysis of the identified metabolites.

**Figure 8:**
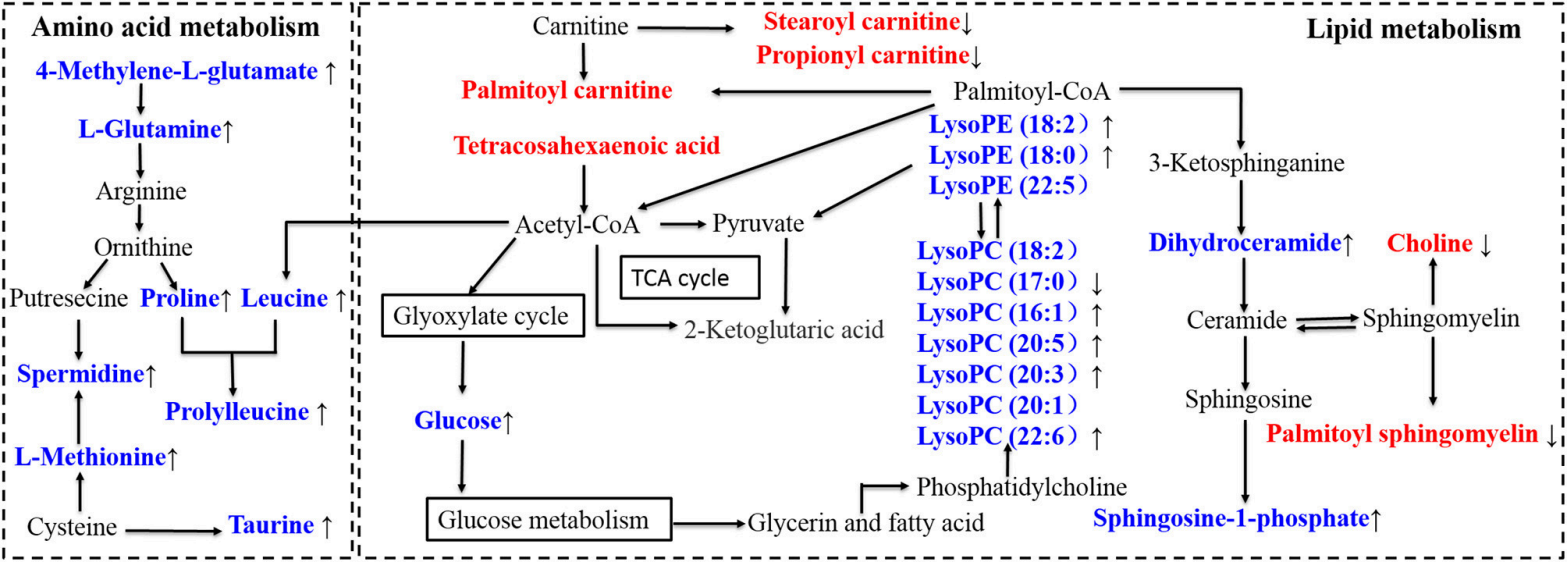
Disturbed metabolic pathway network in sepsis and the interventional effects of Xuebijing injection. The names marked in red represent up-regulated metabolites in septic rats, and the names marked in blue represent down-regulated metabolites in septic rats. The names marked in black represent undetected metabolites. The metabolites reversed by Xuebijing injection are marked with up and down arrows.

In order to explore the pathogenesis of sepsis, the metabolites were submitted to MetaboAnalyst 3.0 to discover the important and significant metabolic pathways (Xia and Wishart, 2016) (shown in Figure 7). A further biology analysis on the pathways has been made which had significant change, and the results suggested that the most metabolic disorders induced by sepsis mainly involved taurine-hypotaurine metabolism, valine-leucine-isoleucine degradation, nicotinate-nicotinamide metabolism etc. After intervened by XBJ injection, most disordered metabolites were adjusted in reverse, and the drug may play an excellent therapeutic effect in this way. Then, a metabolic network was constructed based on the disturbed metabolites by searching KEGG database (shown in Figure 8), and we described an integrated route which could exhibit the complicated connection between the metabolites clearly.

## Discussion

In this study, the antiseptic activity of XBJ injection was revealed by the plasma metabolomics analysis using UHPLC-Q-Orbitrap HRMS. The results demonstrated that XBJ might exert the therapeutic effects on sepsis through multiple metabolic pathways involved energy metabolism, oxidative stress, and inflammation metabolism. The disturbed metabolic pathways discovered in this work were valuable for elucidating the therapeutic mechanism of XBJ.

### Metabolism involved energy

The meatabolic abnormalities of amino acid commonly occured in wasting disease such as sepsis. In this study, the disordered pathways involved amino acid metabolism mainly included valine, leucine, and isoleucine degration, cysteine and methionine metabolism, arginine and proline metabolism, and so on. The amino acid metabolites in the selected pathways all showed an obvious downward trend in the CLP group. An analysis of physiology found that the rats body would experience a state of high power and high metabolism after sepsis occurred. As an energy storage unit, the amino acid (such as Proline, Methionine, and Leucine) would undergo an excessive consumption in sepsis and the level could reflect a dramatically decline. Although the degradation of the amino acid played an important role on maintaining the energy balance and this reflected an automatic compensation mechanism of the life body, the lower concentration of these amino acids could result in increased levels of oxidative stress, which would deteriorate the disease condition (Su et al., 2015). However, in XBJ treatment group, the condition reversed and the amino acid levels increased, which could stabilize the metabolic pathways and weaken the tissue damage. This phenomena may be caused by the condition that glucose and the different gradients in XBJ injection were likely to be preferentially oxidized to provide energy than the other energy material(such as amino acid, protein, and fat) in the body. By this way, the body's energy became sufficient, the amino acid consumption decreased and the plasma level increased significantly. Therefore, the amino acid metabolism began to get it right slowly.

In the process of biological cells energy metabolism, carnitine was considered as an important medium and transported the fatty acids from cytoplasm to mitochondria. Therefore, in order to maintain the energy supplement, carnitine would show a high level in the sepsis period and this had been proved in the experiment results (such as Propionyl carnitine, Palmitoyl carnitine, and Stearoyl carnitine). While, as was described above, this state of energy deficiency would be alleviated after the administration of XBJ injection, then the fatty acids transporting activity came down and the concentration level of carnitine transporter would cut down automatically. In the result of XBJ group, the carnitine level actually had a clear decline, and XBJ could supply energy to satisfy the body metabolism to some extent.

### Metabolism involved oxidative stress

Multiple organ dysfunction syndrome (MODS) induced by sepsis is an important cause of high mortality for patients (Zimbler and Campbel, 2004). Among the numerous and complex mechanisms lead to MODS, oxidative stress injury is one of the crucial influence factors. When sepsis occurs, endotoxins and ischemia-reperfusion injury can activate NADPH oxidase and xanthine oxidase which could result in generation of the reactive oxygen species (ROS) and oxidative/antioxidant disorder, then finally lead to the oxidative stress injury of tissue cells (Lee and Hüttemann, 2014). Hence, it is necessary to pay attention to the oxidative stress injury in sepsis (Yao et al., 2015).

In pathway and importance ranking analysis, it was discovered that taurine and hypotaurine metabolism is a distinct and influential pathway. Taurine is regarded as a kind of abundant free amino acid in mammals and plays an extremely important role in some essential biological processes such as maintenance of calcium homeostasis, bile acid conjugation and membrane stabilization (Marcinkiewicz and Kontny, 2014). At present, increasing evidences show that oxidative stress and inflammation are highly related, which are regarded as essential partners that appear simultaneously and interact each other in various pathological conditions (Li et al., 2016). Many studies suggested that taurine had obvious antioxidant and anti-inflammatory function to maintain the physiological activities of cells and tissues (Ripps and Shen, 2012). The result showed that the level of taurine declined sharply and the rats showed severe inflammation in sepsis model. The main mechanism led to the reduce of taurine was the cells internal flow, the excessive consumption and the lack of synthesize substrate (Wang et al., 2016). This disordered metabolic pathway might have important influence on the development of sepsis. Interestingly, results showed that the taurine level had a prominent increase after giving the treatment of XBJ injection and the levels of inflammatory cytokine dropped significantly. Then we tried to explain how did the drug help taurine to fight with the oxidative stress and elevate its plasma level. The therapeutic material basis was found by analyzing the composition of XBJ injection in our previous study (Zuo et al., 2017), both salvianolic acid from Salvia miltiorrhiza and gallic acid from Radix Paeoniae Rubra exerted a good antioxidant effect (Chen et al., 2013; Nabavi et al., 2016). With the synergistic effect of the active compounds in medicine, taurine could act out an anti-oxidative stress effect to its maximum extent and come with the lower self-depletion in sepsis. Therefore, XBJ might play a role of antioxidant and enhance the taurine level simultaneously to decrease tissue damage and delay the disease progression.

### Metabolism involved inflammation

In the pathway analysis result, it is clear that sphingolipid metabolism occupied an important position. Sphingolipid is basic lipids consisting of a sphingoid framework that is N-acylated with different fatty acids to form many ceramide species. Progress in a large body of evidence demonstrated that sphingolipid metabolites, particularly sphingosine-1-phosphate (S1P) was a signaling molecule that regulated various range of cellular process that was important in inflammation and inflammatory disorders (Michael and Sarah, 2014). The inflammatory phase in sepsis was characterized by excessive release of vast and different cytokines (Cinel and Opal, 2009). S1P had been proved a good anti-inflammatory effects and this anti-inflammatory mechanism might be weaken in sepsis with low S1P level (Rivera et al., 2008). An important role for S1P in inflammation is the regulation of lymphocyte egress from lymphatic organs into the blood. Interestingly, decreased S1P level in sepsis might lead to the hypo-inflammatory state of the disease, which was characterized with immune-paralysis with accompanying lymphopenia and followed the hyper-inflammatory phase typically (Boomer et al., 2013). In this study, the S1P concentrations of CLP group present a low level, and this was associated with disease severity. In addition, some studies demonstrated that low S1P levels would contribute to the capillary leakage, the impaired tissue perfusion and the organ failure in sepsis since S1P was a regulator on endothelial integrity (Winkler et al., 2015). On the contrary, the S1P concentration of XBJ group present a high level which is concomitant with the lower inflammatory cytokine and the less severe state of the body. Therefore, sphingolipid metabolism was a vital pathway in sepsis, and XBJ injections might have a therapeutic effect by intervening this way.

In the tablet result, a lot of Lysophosphatidylcholines (LysoPCs) were discovered and considered as potential biomarkers. The concentration of the LysoPCs in CLP group had an evident decline relative to Sham group. LysoPCs were products of phospholipase A2 (PLA2) and had a direct role in toxic inflammatory response in different organ systems. In sepsis patients, the LysoPCs level in plasma had significantly reduced and systemic treatment with LysoPCs was verified as the therapeutic in rodent models of sepsis (Timothy et al., 2008). The LysoPCs was found to act as uncompetitive product inhibitors of the plasma secreted PLA2 enzymes and thus providing a feedback mechanism. In CLP group, the LysoPCs level was low and the inflammatory response was intense. While in XBJ group, the inflammatory state had been alleviated and accompanied with the high metabolites level. Hence, XBJ injections might play a role on PLA2 enzymes activities and LysoPCs levels to have a further influence on anti-inflammatory.

## Conclusion

This study could give a preliminary exploration on the significant biologically relevant metabolic changes caused by CLP-induced rat sepsis and the underlying mechanisms of XBJ. According to the experiment results, most of disordered biomarkers were adjusted by XBJ in reverse to maintain the balance of the involved pathways. This study indicated that UHPLC-Q-Orbitrap HRMS based metabolomics can provide a scientific and powerful tool for elucidating the energy supply, anti-oxidation, and anti-inflammatory function of XBJ in treating sepsis model rats and exploring the therapeutic mechanisms of the drug on sepsis patients in clinic. Furthermore, it may be useful for obtaining a better understanding on TCM pharmacological mechanism and providing a deeper insight into the theory explanation of TCM through this way.

## Author contributions

LZu, LZh, ZS, and XZ: designed the research, conducted performed the majority of the experiment, and revised the manuscript; TX, ZL, YS, and LL: assisted on supported several experimental performances and deal with the statistical data; JK, GG, and SD: supervised the research and revised the manuscript. All authors approved the final version to be published.

### Conflict of interest statement

The authors declare that the research was conducted in the absence of any commercial or financial relationships that could be construed as a potential conflict of interest.

## Abbreviations

XBJ: Xuebijing
CLP: cecal ligation and puncture
SIRS: systemic inflammatory response syndrome
MODS: Multiple Organ Dysfunction Syndrome
TCM: Traditional Chinese Medicine
SFDA: State Food and Drug Administration
UHPLC-Q-Orbitrap HRMS: ultra performance liquid chromatography-quadrupole/orbitrap high resolution mass spectrometry
HESI: heated electrospray ionization
ELISA: Enzyme-Linke Immuno Sorbent Assay
PCA: principal component analysis
OPLS-DA: orthogonal partial least square discrimination analysis
VIP: Variable Importance in Projection
S1P: sphingosine-1-phosphate
LysoPCs: Lysophosphatidylcholines
PLA2: phospholipase A2.
